# Levothyroxine Treatment and Cardiovascular Outcomes in Older People With Subclinical Hypothyroidism: Pooled Individual Results of Two Randomised Controlled Trials

**DOI:** 10.3389/fendo.2021.674841

**Published:** 2021-05-20

**Authors:** Laurien E. Zijlstra, J. Wouter Jukema, Rudi G. J. Westendorp, Robert S. Du Puy, Rosalinde K. E. Poortvliet, Patricia M. Kearney, Linda O’Keeffe, Olaf M. Dekkers, Manuel R. Blum, Nicolas Rodondi, Tinh-Hai Collet, Terence J. Quinn, Naveed Sattar, David J. Stott, Stella Trompet, Wendy P. J. den Elzen, Jacobijn Gussekloo, Simon P. Mooijaart

**Affiliations:** ^1^ Department of Cardiology, Leiden University Medical Center, Leiden, Netherlands; ^2^ Department of Public Health, University of Copenhagen, Copenhagen, Denmark; ^3^ Center for Healthy Aging, University of Copenhagen, Copenhagen, Denmark; ^4^ Department of Public Health and Primary Care, Leiden University Medical Center, Leiden, Netherlands; ^5^ School of Public Health, University College Cork, Cork, Ireland; ^6^ Department of Endocrinology and Metabolic Disorders, Leiden University Medical Center, Leiden, Netherlands; ^7^ Department of General Internal Medicine, Inselspital, Bern University Hospital, University of Bern, Bern, Switzerland; ^8^ Institute of Primary Health Care (BIHAM), University of Bern, Bern, Switzerland; ^9^ Service of Endocrinology, Diabetology, Nutrition and Therapeutic Education, Geneva University Hospitals, Geneva, Switzerland; ^10^ The Academic Section of Geriatric Medicine, Institute of Cardiovascular and Medical Sciences, University of Glasgow, Glasgow, United Kingdom; ^11^ BHF Glasgow Cardiovascular Research Centre, Faculty of Medicine, Glasgow, United Kingdom; ^12^ Institute of Cardiovascular and Medical Sciences, College of Medical, Veterinary and Life Sciences, University of Glasgow, Glasgow, United Kingdom; ^13^ Department of Internal Medicine, Section of Gerontology and Geriatrics, Leiden University Medical Center, Leiden, Netherlands; ^14^ Department of Clinical Chemistry and Laboratory Medicine, Leiden University Medical Center, Leiden, Netherlands; ^15^ Institute for Evidence-based Medicine in Old Age (IEMO), Leiden, Netherlands

**Keywords:** cardiovascular disease, levothyroxine, randomised controlled trial, subclinical hypothyroidism, older adults

## Abstract

**Background:**

The cardiovascular effects of treating older adults with subclinical hypothyroidism (SCH) are uncertain. Although concerns have been raised regarding a potential increase in cardiovascular side effects from thyroid hormone replacement, undertreatment may also increase the risk of cardiovascular events, especially for patients with cardiovascular disease (CVD).

**Objective:**

To determine the effects of levothyroxine treatment on cardiovascular outcomes in older adults with SCH.

**Methods:**

Combined data of two parallel randomised double-blind placebo-controlled trials TRUST (Thyroid hormone Replacement for Untreated older adults with Subclinical hypothyroidism – a randomised placebo controlled Trial) and IEMO80+ (the Institute for Evidence-Based Medicine in Old Age 80-plus thyroid trial) were analysed as one-stage individual participant data. Participants aged ≥65 years for TRUST (n=737) and ≥80 years for IEMO80+ (n=105) with SCH, defined by elevated TSH with fT4 within the reference range, were included. Participants were randomly assigned to receive placebo or levothyroxine, with titration of the dose until TSH level was within the reference range. Cardiovascular events and cardiovascular side effects of overtreatment (new-onset atrial fibrillation and heart failure) were investigated, including stratified analyses according to CVD history and age.

**Results:**

The median [IQR] age was 75.0 [69.7–81.1] years, and 448 participants (53.2%) were women. The mean TSH was 6.38± SD 5.7 mIU/L at baseline and decreased at 1 year to 5.66 ± 3.3 mIU/L in the placebo group, compared with 3.66 ± 2.1 mIU/L in the levothyroxine group (p<0.001), at a median dose of 50 μg. Levothyroxine did not significantly change the risk of any of the prespecified cardiovascular outcomes, including cardiovascular events (HR 0.74 [0.41–1.25]), atrial fibrillation (HR 0.69 [0.32–1.52]), or heart failure (0.41 [0.13–1.35]), or all-cause mortality (HR 1.28 [0.54–3.03]), irrespective of history of CVD and age.

**Conclusion:**

Treatment with levothyroxine did not significantly change the risk of cardiovascular outcomes in older adults with subclinical hypothyroidism, irrespective of a history of cardiovascular disease and age.

**Clinical Trial Registration:**

[ClinicalTrials.gov], identifier [NCT01660126] (TRUST); Netherlands Trial Register: NTR3851 (IEMO80+).

## Introduction

Subclinical hypothyroidism (SCH) is a common condition in older adults, with a prevalence between 8% and 18% (1). SCH is defined by elevated levels of thyroid stimulating hormone (TSH) with free thyroxine (fT4) within the reference range. Patients with SCH are mostly asymptomatic, although SCH is a possible contributor to various health problems including cardiovascular diseases (CVD) (2). The cardiovascular effect of treating older adults with SCH is uncertain.

The cardiovascular system is sensitive to changes in thyroid hormone concentrations due to thyroid hormone receptors in myocardial and vascular endothelial tissues (3). Associations have been found between SCH and an increase in the number of cardiovascular risk factors (4). In addition, meta-analyses of prospective studies showed that SCH was associated with an increased risk of heart failure, major adverse cardiovascular events (MACE) and cardiovascular death (2, 5–7). Although associations have been found between SCH and CVD, data are limited and conflicting regarding the effect of treatment with levothyroxine on cardiovascular outcomes (3). Large randomised controlled trials (RCT) investigating especially cardiovascular outcomes in older patients are limited and most often investigated surrogate markers of CVD, such as cardiovascular risk factors (8) or cardiac function and structure (9–11). On the one hand, concerns have been raised regarding a potential increase in cardiovascular side effects from thyroid hormone replacement, such as atrial fibrillation and heart failure. On the other hand, undertreatment may increase the risk of cardiovascular events, especially for patients with CVD or older age.

Two recent RCTs, namely TRUST (Thyroid hormone Replacement for Untreated older adults with Subclinical hypothyroidism – a randomised placebo controlled Trial) and IEMO80+ (the Institute for Evidence-Based Medicine in Old Age 80-plus thyroid trial), reported the absence of beneficial effect of levothyroxine on thyroid specific quality of life related outcomes in older patients with SCH (12, 13). For the first time combining all data from the two trials, the aim of the present study is to assess the effect of levothyroxine treatment on cardiovascular outcomes in older adults with SCH.

## Materials and Methods

This study is a prespecified combined analysis of the TRUST and IEMO80+ studies. These studies were designed and executed as parallel trials with identical study protocols, both investigating whether levothyroxine provides clinical benefits in older persons with SCH.

An Institutional Review Board approved the studies prior to data collection. Written informed consent was obtained from all participants. Data were analysed as one-stage individual participant data (IPD) of these two randomised double-blind placebo-controlled trials. Detailed description and protocols have been published previously (14, 15). In summary, older participants (≥65 years for TRUST and ≥80 years for IEMO80+) with SCH, diagnosed by elevated TSH levels (4.6 to 19.9 mIU/L), measured on at least two occasions between 3 months and 3 years apart, with fT4 levels within the reference range, were enrolled in Ireland, Scotland, Switzerland and The Netherlands. Participants were randomised in a 1:1 ratio for levothyroxine or placebo, with titration of the levothyroxine dose according to TSH level every 6 to 8 weeks and a mock titration schedule with a similar frequency in the placebo group. The levothyroxine group started with a dose of 50μg daily (or 25μg for participants with weight <50kg or a history of coronary heart disease). Participants were followed up for a minimum of 12 months and a maximum of 36 months between April 2013 and May 2018. The final follow-up was on May 4, 2018.

### Endpoints

The present analysis reports cardiovascular outcomes, including all-cause and cardiovascular mortality, and both cardiovascular events and cardiovascular side effects. Cardiovascular events are fatal and non-fatal cardiovascular events, including acute myocardial infarction, stroke, amputations for peripheral vascular disease, revascularisations for atherosclerotic vascular disease (including for acute coronary syndrome) and heart failure hospitalisations. Cardiovascular side effects of overtreatment include new-onset atrial fibrillation and new-onset heart failure. Secondary outcomes include the cardiovascular parameters blood pressure, heart rate and weight, which were measured as positive signals of TSH change.

### History of Cardiovascular Disease and Age

Stratified analyses were executed for patients with or without a history of CVD at inclusion. CVD was defined as ischemic heart disease (both angina pectoris or myocardial infarction), stroke or transient ischemic attack, heart failure, peripheral vascular disease, revascularization or atrial fibrillation. Furthermore, patients were stratified in the 65 to 80 age range, or ≥80 years old.

### Statistical Analysis

Baseline characteristics are presented as mean ± standard deviation (SD) or median [interquartile range (IQR)] depending on the distribution of data, stratified for history of CVD. Hazard ratios (HR) were obtained from a Cox proportional hazard regression model and were adjusted for country, sex, starting dose of levothyroxine and study, similar to previous publications (14, 15). Results at 12 months and between-group differences were adjusted for country, sex, starting dose of levothyroxine, study (TRUST or IEMO80+) as random effect and baseline levels of the same variable with the use of linear mixed models. Between-group differences are the value in the levothyroxine group minus the value in the placebo group. The efficacy and safety analyses were carried out in a modified intention-to-treat population, which included participants with data on the outcome of interest. The data were analysed using IBM SPSS Statistics, version 23. P-values were considered statistically significant if lower than 0.05. Interaction analyses were performed between treatment and history of CVD and all secondary endpoints.

## Results

**Figure 1:**
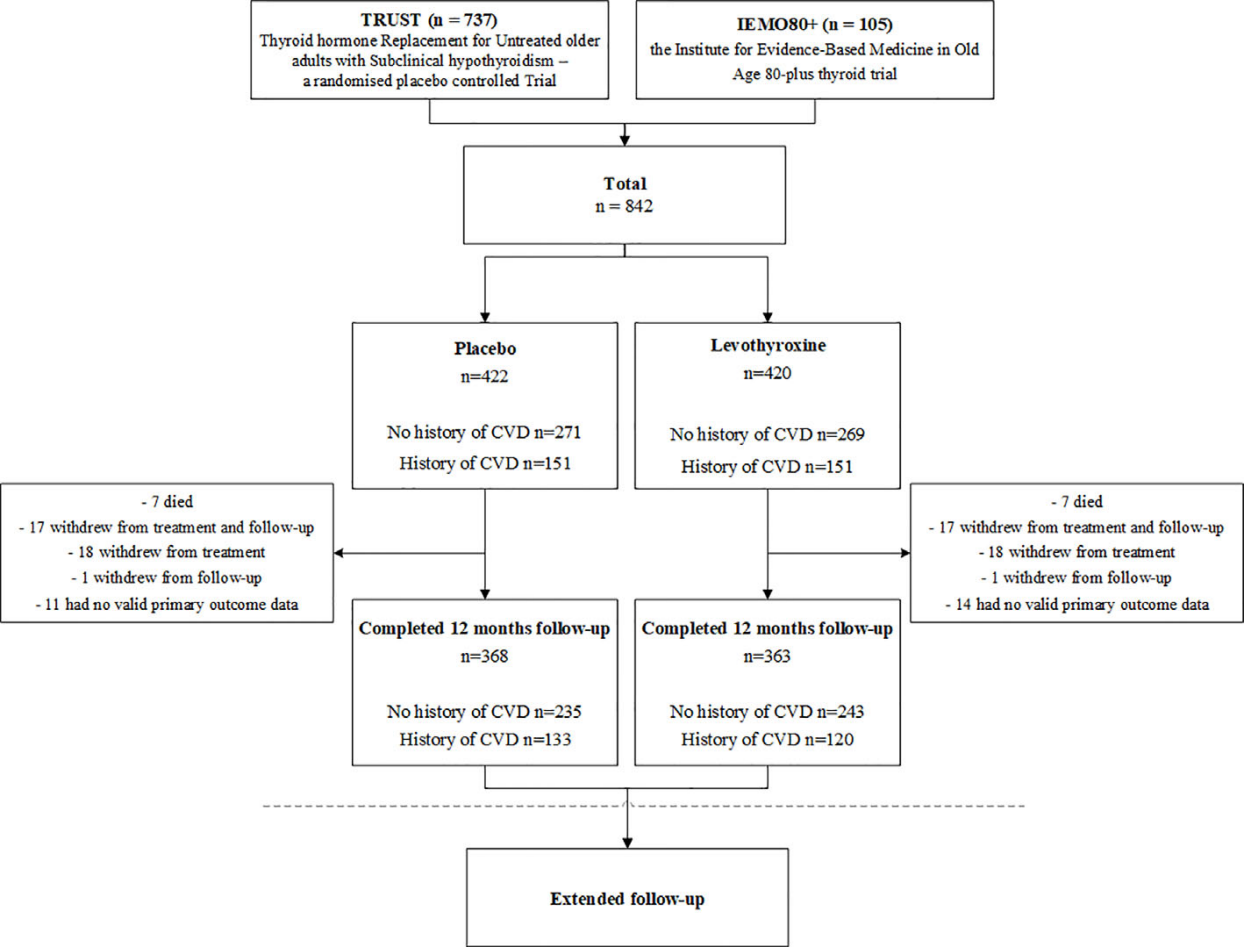
Flowchart study population. Combined data of the TRUST and IEMO80+ trials will be examined as one-stage individual participant data of these two randomised double-blind placebo-controlled parallel group trials. Cardiovascular disease (CVD) is defined as ischemic heart disease, stroke or transient ischemic attack, heart failure, peripheral vascular disease, revascularisation or atrial fibrillation. Median follow-up was 17-months.

In total, all 737 patients from TRUST and all 105 patients from IEMO80+ were included in this combined data-analysis, see **Figure 1**. Of the 842 participants who underwent randomization, 422 were assigned to receive placebo and 420 to receive levothyroxine. For the baseline characteristics see **Table 1**. The median age of the 842 participants was 75.0 [IQR 69.7–81.1] years, with 419 (56.9%) participants older than 80 years. In total, 448 participants (53.2%) were women and 302 (35.9%) had a history of CVD. History of CVD or cardiovascular risk factors did not differ between the placebo or levothyroxine group. Median follow-up was 17 months. A total of 368 participants (87.2%) of the placebo group and 363 (86.4%) of the levothyroxine group completed 12-month follow-up, which did not differ between patients with or without a history of CVD, see **Figure 1**. In total, 194 (23.0%) patients discontinued the trial regimen and 44 (5.2%) withdrew from follow-up. Most participants (83.8%) started with a dose of 50 μg and 16.2% with 25 μg levothyroxine. Of patients with a history of CVD, 58.9% started with a dose of 50 μg levothyroxine and of patients older than 80 79.5%.

**Table 1:** Baseline characteristics (n = 842).

Characteristic	No history of CVD		History of CVD	
	Placebo (n = 271)	Levothyroxine (n = 269)	Placebo (n = 151)	Levothyroxine (n = 151)
Age (years), median [IQR]	72.7 [68.6-79.2]	73.6 [68.9-78.8]	79.9 [73.0-84.5]	76.8 [72.0-81.7]
Female sex, n (%)	162 (59.8)	161 (59.9)	62 (41.1)	63 (41.7)
Caucasian^a^, n (%)	264 (97.4)	264 (98.1)	150 (99.3)	150 (99.3)
Standard housing^b^, n (%)	264 (97.4)	262 (97.4)	142 (94.0)	145 (96.0)
History of cardiovascular disease, n (%)				
Ischemic heart disease^c^			62 (41.1)	63 (41.7)
Stroke or transient ischemic attack			55 (36.4)	33 (21.9)
Peripheral vascular disease			15 (9.9)	20 (13.3)
Revascularisation			46 (30.5)	59 (39.1)
Heart failure			23 (15.2)	15 (9.9)
Atrial fibrillation			53 (35.3)	58 (38.9)
Cardiovascular risk factors, n (%)				
Hypertension	115 (42.8)	133 (49.4)	92 (61.3)	86 (57.0)
Diabetes mellitus	29 (10.7)	40 (14.9)	28 (18.7)	30 (19.9)
Current smoking	25 (9.2)	19 (7.1)	10 (6.6)	13 (8.6)
Former smoking	105 (38.7)	112 (41.6)	76 (50.3)	74 (49.0)
Number of concomitant medicines	3 [1-5]	3 [1-5]	6 [4-6]	6 [4-8]
Clinical parameters				
Body mass index (kg/m^2^)	27.2 ± 4.6	27.8 ± 5.2	28.5 ± 4.5	28.5 ± 5.4
Waist circumference (cm)	95.7 ± 12.9	97.1 ± 12.4	100.8 ± 11.4	100.9 ± 12.4
Blood pressure (mmHg)				
Systolic	142 ± 20	143 ± 18	142 ± 20	140 ± 21
Diastolic	75 ± 12	75 ± 11	73 ± 12	72.3 ± 10
Heart rate (beats per min.)	70.4 ± 10.6	69.1 ± 10.6	68.6 ± 13.0	67.6 ± 12.7
Hand-grip strength (kg)	27.3 ± 10.5	27.2 ± 10.3	27.3 ± 11.9	28.5 ± 10.3
Thyroid function^d^				
Thyrotropin (mIU/liter)	6.4 ± 2.1	6.5 ± 2.1	6.2 ± 1.8	6.2 ± 1.7
Median	5.7 [5.1-7.0]	5.7 [5.2-7.0]	5.7 [5.0-6.8]	5.7 [5.0-6.8]
Free thyroxine (pmol/liter)	13.1 ± 1.9	13.4 ± 2.0	14.0 ± 2.0	13.7 ± 2.2
Quality of life^e^				
Hypothyroid Symptoms score	15.4 ± 16.9	17.5 ± 19.2	21.0 ± 20.4	18.9 ± 18.2
Tiredness score	23.0 ± 18.2	25.7 ± 20.7	29.6 ± 22.5	25.5 ± 20.6
EQ-5D descriptive index	0.855 ± 0.18	0.847 ± 0.18	0.804 ± 0.20	0.819 ± 0.22
EQ visual-analogue scale score	77.6 ± 15.9	79.2 ± 15.2	73.3 ± 15.3	76.0 ± 15.1

Values are mean ± standard deviation (SD) or median [interquartile range (IQR)]. Cardiovascular disease (CVD) was defined as ischemic heart disease (both angina pectoris or myocardial infarction), stroke or transient ischemic attack, heart failure, peripheral vascular disease, revascularization or atrial fibrillation. ^a^Race was reported by the patient. ^b^Standard housing was defined as non-sheltered community accommodation. By contrast, sheltered housing is purpose built grouped housing for older persons, often with an on-site manager or warden. ^c^Ischemic heart disease was defined as a history of angina pectoris or previous myocardial infarction. ^d^ To convert the values for free thyroxine to nanograms per deciliter, divide by 12.87. ^e^The Hypothyroid Symptoms score and the Tiredness score from the Thyroid-Related Quality of Life Patient-Reported Outcome (ThyPRO) questionnaire are each assessed on a scale from 0 to 100, with higher scores indicating more symptoms and tiredness, respectively. The minimum clinically important difference for each score has been estimated as 9 points. The EuroQoL [EQ] Group 5-Dimension Self-Report Questionnaire (EQ-5D) scores included both the EQ5D descriptive index (on a scale from −0.59 to 1.00) and the score on the EQ visual-analogue scale (on a scale from 0 to 100); higher scores on each scale indicate better quality of life.

### Thyroid Function

The mean ± SD TSH was 6.38 ± 5.7 mIU/L at baseline, and decreased at 1 year to 5.66 ± 3.3 mIU/L in the placebo group, compared with 3.66 ± 2.1 mIU/L in the levothyroxine treated group (p<0.001), at a median dose of 50 μg. TSH did not differ significantly at baseline or at 12 months between patients with or without a history of CVD (p-interaction=0.31), see **Table 2**.

**Table 2:** Thyroid function and cardiovascular parameters at 12 months for patients with or without a history of cardiovascular disease*.

Variable	No history of CVD					History of CVD						
	Baseline		At 12 months			Baseline		At 12 months			Interaction	
	Placebo(n = 271)	Levothyroxine(n = 269)	Placebo(n = 235)	Levothyroxine (n = 243)	Difference(95% CI)	Placebo(n = 151)	Levothyroxine(n = 151)	Placebo(n = 133)	Levothyroxine(n = 120)	Difference(95% CI)	p-value	
Thyrotropin (mIU/L)	6.4 ± 0.1	6.5 ± 0.1	5.6 ± 0.2	3.5 ± 0.1	−2.12 (−2.49 to −1.76)	6.2 ± 0.2	6.2 ± 0.1	5.5 ± 0.2	3.8 ± 0.2	−1.63 (−2.17 to −1.11)	0.31	
Median [IQR]	5.8 [5.1 to 7.0]	5.7 [5.2 to 7.0]	4.9 [4.6 to 6.6]	3.2 [2.4 to 4.2]		5.2 [5.0 to 6.8]	5.2 [5.0 to 6.8]	4.9 [3.9 to 6.4]	3.5 [2.7 to 4.4]			
Range	4.6 to 17.6	4.6 to 17.6	0.1 to 46.0	0.03 to 15.9		4.6 to 17.6	4.6 to 14.2	1.9 to 18.0	0.8 to 15.4			
**Cardiovascular parameters**											
Systolic blood pressure (mm Hg)	142 ± 1.2	143 ± 1.1	139 ± 1.2	140 ± 1.1	0.96 (−1.67 to 3.59)	142 ± 1.6	140 ± 1.7	139 ± 1.7	137 ± 1.9	−1.05 (−5.23 to 3.13)	0.40	
Diastolic blood pressure (mm Hg)	75 ± 0.7	75 ± 0.7	74 ± 0.7	74 ± 0.7	0.46 (−1.11 to 2.03)	73 ± 1.0	72 ± 1.0	71 ± 1.1	69 ± 1.2	–1.16 (–3.62 to 1.29)	0.28	
Heart rate (beats per minute)	70.4 ± 0.6	69.1 ± 0.6	70.1 ± 0.7	69.3 ± 0.7	1.07(−0.50 to 2.63)	68.6 ± 1.1	67.6 ± 1.0	68.7 ± 1.2	67.2 ± 1.2	–0.78 (–3.12 to 1.56)	0.17	
Weight (kg)	74.9 ± 0.9	76.3 ± 0.9	75.0 ± 0.9	76.3 ± 0.9	0.16 (−0.36 to 0.69)	79.3 ± 1.2	80.1 ± 1.4	79.7 ± 1.3	80.6 ± 1.5	0.27 (−0.67 to 1.21)	0.83	

Values are mean ± standard error (SE). CI, confidence interval; CVD, cardiovascular disease. Results at 12 months and between-group differences are adjusted for stratification variables (country, sex, starting dose of levothyroxine and study as random effect) and baseline levels of the same variable with the use of linear mixed models. Between-group differences are the value in the levothyroxine group minus the value in the placebo group. Interaction analyses were performed between treatment and history of CVD and all endpoints. *CVD was defined as ischemic heart disease (both angina pectoris or myocardial infarction), stroke or transient ischemic attack, heart failure, peripheral vascular disease, revascularization or atrial fibrillation.

### Cardiovascular Outcomes

**Figure 2:**
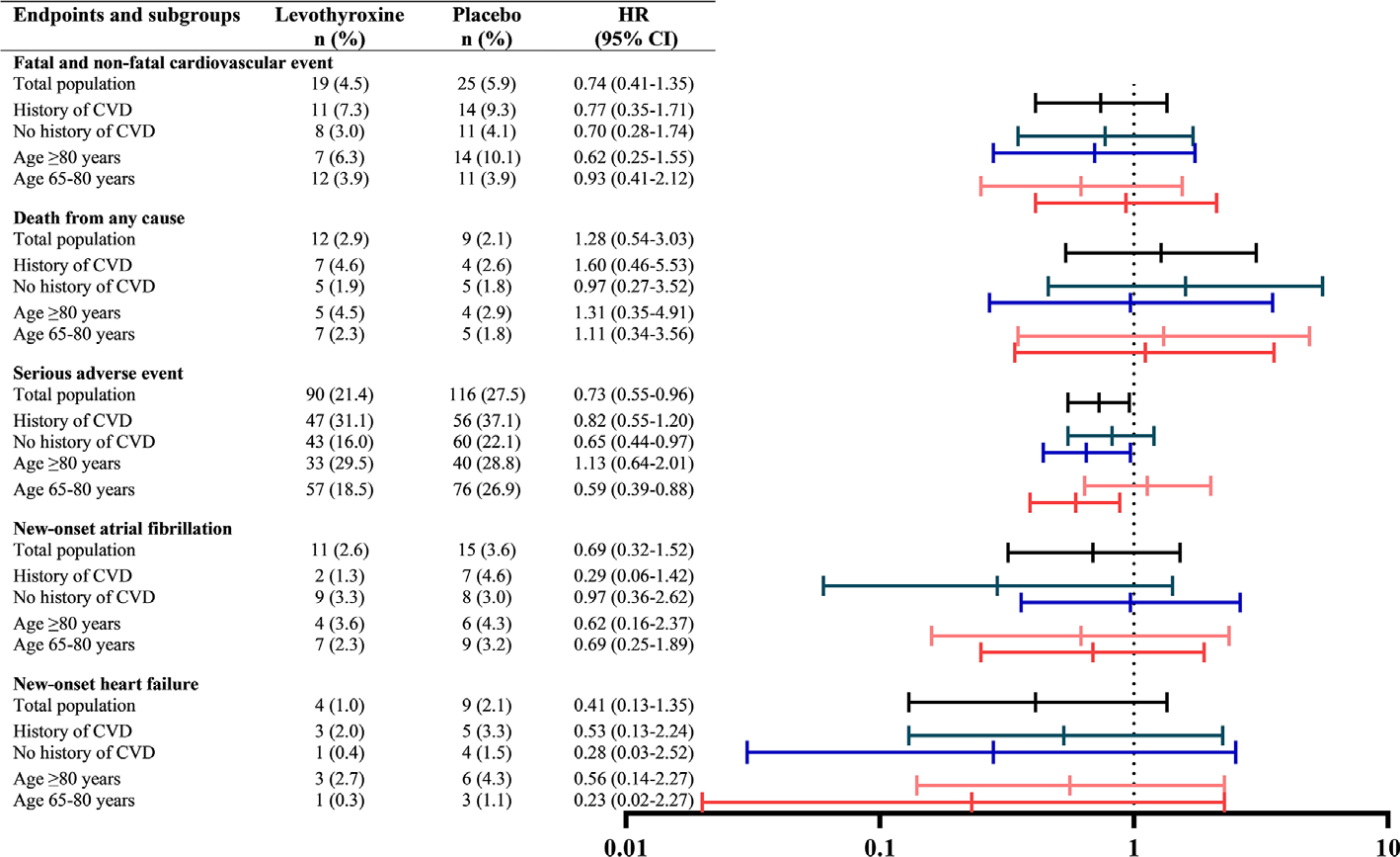
Cardiovascular outcomes stratified for history of cardiovascular disease and age. Cardiovascular disease (CVD) is defined as ischemic heart disease, stroke or transient ischemic attack, heart failure, peripheral vascular disease, revascularisation or atrial fibrillation. Hazard ratios for treatment were obtained from a Cox proportional hazard regression model predicting and were adjusted for study, country, sex and starting dose of levothyroxine.

In total, 44 (5.2%) fatal and non-fatal cardiovascular events occurred after a median follow-up of 17 months, which did not significantly differ between placebo and levothyroxine with a HR comparing treatment to placebo of 0.74 (0.41 to 1.35). Comparing cardiovascular side effects of overtreatment risk of new-onset atrial fibrillation was associated with levothyroxine treatment was HR 0.69 (0.32 to 1.52) and HR of new-onset heart failure was 0.41 (0.13 to 1.35). Furthermore, in total, 21 (2.5%) deaths from any cause occurred (of which 4 cardiovascular deaths) with a HR for levothyroxine treatment of 1.28 (0.54 to 3.03). **Figure 2** shows a forest plot of all cardiovascular outcomes, comparing placebo to levothyroxine stratified by history of CVD and age, showing that levothyroxine did not significantly change the risk of any of the cardiovascular outcomes, irrespective of CVD history or age (p for interaction all >0.10).

No clinically relevant or statistically significant adjusted differences between levothyroxine and placebo were found at 12 months for blood pressure, heart rate and weight (**Table 2**). Outcomes did not differ between patients with or without a history of CVD (p for interaction all >0.10).

## Discussion

In this prespecified combined analysis of the TRUST and IEMO80+ trials, treatment with levothyroxine did not increase or decrease the risk of cardiovascular outcomes significantly in older adults with SCH, irrespective of CVD history and age.

In older patients with SCH, the current European and United States guidelines recommend no routine thyroid hormone therapy (16, 17). Especially in older people, treatment should be individualised, gradual and closely monitored. In the oldest old subjects, defined as >80 years old, SCH should be carefully followed with a wait-and-see strategy, generally avoiding hormonal treatment (16). The outcomes of both TRUST and IEMO80+ support this wait-and-see strategy as they showed no consistent beneficial effect of levothyroxine on quality of life, in both older (TRUST) and oldest old subjects (IEMO80+) (12, 13). Although experts have pointed out the need, before the TRUST and IEMO80+ studies, randomised trials investigating hard cardiovascular endpoints were lacking (18). Hence we sought to answer the question whether undertreatment may cause cardiovascular events or treatment may cause cardiovascular side effects. We found no significant adjusted differences in cardiovascular parameters and neutral results for all cardiovascular outcomes with wide confidence intervals, although all point estimates were favourable for levothyroxine treatment. Therefore, we found no evidence to support any major short to medium term harmful effect on cardiovascular events of levothyroxine treatment for subclinical hypothyroidism in older people, including in those with known prior cardiovascular disease.

Taken together, our finding that treatment with levothyroxine did not change the risk of all cardiovascular outcomes in older adults with SCH is of incremental value to the limited existing literature. We showed that when treatment with levothyroxine is indicated on an individual basis, treatment should not be initiated especially to prevent cardiovascular events, nor should it be withheld because of potential cardiovascular side effects, irrespective of CVD history. Provided that treatment should be carefully monitored and titrated over time, as was in the trials.

### Strengths and Limitations

This is a unique combined data analysis of the two largest RCTs to date investigating cardiovascular outcomes in older patients with SCH. Some limitations should be mentioned.

First, it was initially planned in both TRUST and IEMO80+ that cardiovascular events were to be a primary outcome together with thyroid-specific quality of life. Owing to delays and difficulties in recruitment this was changed as it became apparent that both studies would be underpowered for this aspect (13). However, the studies combined enabled the largest data analysis thus far regarding this subject and it is unlikely that a similar experiment will be successful in the near future, especially not a large one. Overall incidence of cardiovascular outcomes after a median follow-up of 17 months in the patients with SCH was still relatively low, only 44 (5.2%) patients had a fatal or nonfatal cardiovascular event. However, 17 months is still relatively short, and does not exclude a substantial cardiovascular 10-year risk. Second, the limited power hampered us to further stratify according to history of CVD and to distinguish between patients with ischemic heart disease, patients with heart failure or patients with vascular disease elsewhere in the body (e.g. cerebrovascular or peripheral artery disease). Furthermore, of the 252 patients with a history of CVD, only 38 subjects had a history of heart failure. Third, of all included older participants, only 251 (29.8%) defined as the oldest old (≥80 years old). Fourth, mean TSH level was not very high at baseline in the total population (6.4 ± 2.0). Fifth, with respect to ethnicity the study population was predominantly white (98%).

### Conclusions

Treatment with levothyroxine did not significantly change the risk of cardiovascular outcomes in older adults with subclinical hypothyroidism, irrespective of a history of CVD.

## Data Availability Statement

Who can access the data: The authors welcome proposals for joint use of the study data after the planned publications of the study data have been completed. Types of analyses: For any purpose, after review and approval from a board of Principle Investigators. Mechanisms of data availability: Data will be made available with investigator support, with a signed data access agreement, after approval of a proposal.

## Ethics Statement

For the UK, the study was approved by the Multicentre Research Ethics Committee (A) and the MHRA, with co-sponsors NHS Greater Glasgow and Clyde and the University of Glasgow. For the Netherlands, the study was approved by the Medical Ethical Committee on Research Involving Human Subjects (CCMO). In Switzerland, the study was approved by the Bern and Lausanne ethical boards and by Swissmedic, the Swiss competent authority for drugs. In Ireland, the study was approved by the Clinical Research Ethics Committee, Cork and by the Health Products Regulatory Authority (formerly known as the Irish Medicines Board). The patients/participants provided their written informed consent to participate in this study.

## Author Contributions

SM and LZ had full access to all the data in the study and take responsibility for the integrity of the data and the accuracy of the data analysis. Concept and design: JJ, RW, PK, OD, NR, TQ, DS, WE, JG, and SM. Acquisition, analysis, or interpretation of data: all authors. Drafting of the manuscript: LZ, JJ, and SM. Critical revision of the manuscript for important intellectual content: all authors. Statistical analysis: LZ. Obtained funding: RW, PK, NR, DS, JG, SM. Administrative, technical, or material support: LZ, JJ, RW, RP, KP, PK, OD, NR, WE, JG, and SM. Study supervision: JJ, RW, OD, NR, JG, and SM. All authors contributed to the article and approved the submitted version.

## Funding

The TRUST trial was supported by a research grant (278148) from the European Union FP7-HEALTH-2011 program and by grants from the Swiss National Science Foundation (SNSF 320030-150025 and 320030-172676; to NN, P2BEP3_165409), and the Swiss Heart Foundation and an investigator-driven grant of Velux Stiftung (974a, to NR). Study medication (levothyroxine and matching placebo) was supplied free of charge by Merck KGaA. The IEMO trial was supported by research grant (627001001) from ZonMw under the ZonMw programme Evidence-based Medicine in Old age and by grants from the Swiss National Science Foundation (SNSF 320030-150025 and 320030-172676 to NR). T-HC’s research is funded by the Swiss National Science Foundation (grant no PZ00P3-167826), the Swiss Society of Endocrinology and Diabetes, the Leenaards Foundation, and the Vontobel Foundation. The funders had no role in the design and conduct of the study, collection, management, analysis, and interpretation of the data, preparation, review, or approval of the manuscript, and decision to submit the manuscript for publication.

## Conflict of Interest

SM reported receiving grants from ZonMW and nonfinancial support from Merck during the conduct of the study. RP reported receiving grants from European Union FP7 and ZonMw (627001001) and nonfinancial support from Merck KGaA during the conduct of the study. DS reported receiving grants from European Union FP7 and nonfinancial support from Merck Serono during the conduct of the study. NR reported receiving grants from the Swiss National Science Foundation and the Velux Foundation during the conduct of the study. KP reported receiving grants from Netherlands Organisation for Health Research and Development (ZonMw) and the European Union FP7-HEALTH-2011 programme during the conduct of the study. T-HC reported receiving grants from the Swiss National Science Foundation during the conduct of the study. JG reported receiving grants from European Union FP7 and ZonMw (627001001) and nonfinancial support from Merck KGaA during the conduct of the study.

The remaining authors declare that the research was conducted in the absence of any commercial or financial relationships that could be construed as a potential conflict of interest.

## Abbreviations

95% CI, 95% confidence interval; EQ-5D, EuroQol Group 5-Dimension Self-Report Questionnaire; fT4, free thyroxine 4; HR, hazard ratio; IEMO80+, the Institute for Evidence-Based Medicine in Old Age 80-plus thyroid trial; IPD, individual participant data; RCT, randomised controlled trial; SCH, subclinical hypothyroidism; SD, standard deviation; SE, standard error; TRUST, Thyroid hormone Replacement for Untreated older adults with Subclinical hypothyroidism – a randomised placebo controlled Trial; TSH, thyroid stimulating hormone; ThyPRO, Thyroid Related Quality-of-Life Patient-Reported Outcome measure.
